# Myc Enforces Overexpression of *EZH2* in Early Prostatic Neoplasia via Transcriptional and Post-transcriptional Mechanisms

**DOI:** 10.18632/oncotarget.327

**Published:** 2011-09-19

**Authors:** Cheryl M. Koh, Tsuyoshi Iwata, Qizhi Zheng, Carlise Bethel, Srinivasan Yegnasubramanian, Angelo M. De Marzo

**Affiliations:** ^1^Department of Pathology, The Johns Hopkins University, School of Medicine, Baltimore, MD, USA; ^2^Department of Urology, The Johns Hopkins University, School of Medicine, Baltimore, MD, USA; ^3^Department of Oncology, The Johns Hopkins University, School of Medicine, Baltimore, MD, USA; ^4^The Sidney Kimmel Comprehensive Cancer Center at Johns Hopkins, The Johns Hopkins University, School of Medicine, Baltimore, MD, USA; ^5^The Brady Urological Research Institute, The Johns Hopkins University, School of Medicine, Baltimore, MD, USA

**Keywords:** Myc, EZH2, prostate cancer

## Abstract

EZH2 is part of the PRC2 polycomb repressive complex that is overexpressed in multiple cancer types and has been implicated in prostate cancer initiation and progression. Here, we identify EZH2 as a target of the MYC oncogene in prostate cancer and show that MYC coordinately regulates EZH2 through transcriptional and post-transcriptional means. Although prior studies in prostate cancer have revealed a number of possible mechanisms of EZH2 upregulation, these changes cannot account for the overexpression EZH2 in many primary prostate cancers, nor in most cases of high grade PIN. We report that upregulation of Myc in the mouse prostate results in overexpression of *EZH2* mRNA and protein which coincides with reductions in miR-26a and miR-26b, known regulators of EZH2 in some non-prostate cell types, albeit not in others. Further, in human prostate cancer cells, Myc negatively regulates miR-26a and miR-26b via direct binding to their parental Pol II gene promoters, and forced overexpression of miR-26a and miR-26b in prostate cancer cells results in decreased EZH2 levels and suppressed proliferation. In human clinical samples, miR-26a and miR-26b are downregulated in most primary prostate cancers. As a separate mechanism of EZH2 mRNA upregulation, we find that Myc binds directly to and activates the transcription of the *EZH2* promoter. These results link two major pathways in prostate cancer by providing two additional and complementary Myc-regulated mechanisms by which EZH2 upregulation occurs and is enforced during prostatic carcinogenesis. Further, the results implicate EZH2-driven mechanisms by which Myc may stimulate prostate tumor initiation and disease progression.

## INTRODUCTION

EZH2 is a histone lysine methyltransferase that is part of the PRC2 polycomb repressive complex. It catalyzes the tri-methylation of histone 3 on lysine 27 (H3K27me3), which is involved in chromatin remodeling and the silencing of genes, including homeobox genes involved in differentiation [1-4]. EZH2 is overexpressed during prostate cancer progression [5, 6] and promotes proliferation, invasion, tumor formation, and metastasis of prostate cancer cells, and is considered to be involved in the progression to advanced disease [7-11]. Prior studies indicated that at least three separate mechanisms could result in EZH2 upregulation in prostate cancer. Saramäki et al. found that a region on chromosome 7q36.1 harboring *EZH2* is amplified in approximately 20% of castrate resistant prostate cancers [8]. More recently, Varambally et al. used a bioinformatics approach to nominate miR-101 as a potential microRNA that can target *EZH2* mRNA for silencing [5]. miR-101 was shown to specifically target EZH2 for down regulation in prostate cancer cells, and the *miR-101* locus was deleted in 37.5% of localized prostate cancers and 66.7% of castrate-resistant metastatic prostate cancer cases. Additionally, Yu et al. and Kunderfranco et al. reported that the ETS gene family members ERG and ESE3 could activate and repress EZH2 transcription, respectively [12, 13]. Interestingly, the original study by Varambally et al. documented upregulation of EZH2 in the majority of all prostate cancer cases, as well as in the majority of all high grade PIN, a key prostate cancer precursor lesion [6]. The findings published to date, therefore, can explain EZH2 upregulation in only a subset of cases of primary and metastatic prostate cancer, and the mechanisms by which EZH2 is upregulated in PIN lesions and in a subset of early carcinomas have not been fully elucidated. Thus, we hypothesized that additional mechanisms are likely operative that induce EZH2 upregulation in prostate cancer, at least during the early phases of the disease.

MYC is one of the most commonly overexpressed oncogenes in cancer [14]. In prostate cancer, Myc overexpression is a frequent and early event, occurring in lesions of both high grade prostatic intraepithelial neoplasia (PIN), and localized and metastatic prostatic adenocarcinomas, suggesting that Myc contributes to the initiation and progression of the disease [15]. Myc is a pleiotropic transcription factor which is key in controlling the expression of genes involved in DNA replication, protein synthesis, cell cycle progression, cellular metabolism, chromatin structure, differentiation and stem cell fate [14, 16]. It was previously shown that Myc can activate and repress the expression of numerous miRNAs [17-20], hence providing additional mechanisms by which Myc can control gene expression. Studies carried out in lymphoma cells and hepatocelluar carcinoma cells have shown that Myc represses the expression of a number of miRNAs, including miR-26a and miR-26b [17, 21]. Interestingly, the 3′UTR of *EZH2* contains target sites for miR-26a and miR-26b, and EZH2 was shown to be targeted for repression by miR-26a in muscle and lymphoma cells [21, 22], and of miR-26b in HeLa cells [23]. Hence, we hypothesized that Myc may be a key driver of EZH2 overexpression in PIN and prostate cancer lesions via repression of miR-26a and miR-26b. Further, since Myc is a well-known sequence specific transacting factor that binds to promoter regions and regulates transcription of a relatively large number of target genes, we also investigated whether Myc can directly transactivate EZH2 transcription as well.

## MATERIALS AND METHODS

### Animal studies

The experimental protocol was approved by the Animal Care and Use Committee at Johns Hopkins University, and the animals were cared for in accordance with institutional guidelines. The Lo-MYC transgenic mice used in this study were obtained from the Mouse Repository of the National Cancer Institute Mouse Models of Human Cancer Consortium at NCI Frederick, MD, USA, and were maintained as previously described [24].

### Cell Culture

The human prostate cancer cell lines LNCaP, CWR22rv1, DU145 and PC3, were obtained from the American Type Culture Collection (ATCC). MYC-CaP cells were a generous gift from Charles Sawyers [25]. They were maintained at 37°C and 5% CO_2_, and supplemented with RPMI 1640 or DMEM with 10% serum.

### Transfections

Cells were transfected using Oligofectamine or Lipofectamine 2000 (Invitrogen). MYC siRNA pools (Dharmacon, L-003282), siCONTROL Non-Targeting siRNA pool #1 (Dharmacon, D-001810), miRIDIAN Mimic hsa-mir-26a (Dharmacon, C-300499), miRIDIAN Mimic hsa-mir-26b (Dharmacon, C-300501) and miRIDIAN microRNA Mimic Negative Control #1 (Dharmacon CN-001000) were transfected at a final concentration of 50nM.

### Quantitative Real-time PCR

RNA was isolated from cells using miRNeasy kit (Qiagen), and from tissue sections using Trizol (Invitrogen). cDNA was synthesized using the RetroScript kit (Ambion). Quantitative RT-PCR was carried out using iQ SYBR Green Supermix (Biorad). PCR primers are shown in Supplementary Table 1. The relative amount of the gene of interest was determined using the ΔΔ*C*_t_ method, relative to TBP and to the control cells, or matched normal tissue. Mature miR-26a and miR-26b expression was measured by Taqman assay (Applied Biosystems) according to manufacturer's instructions, and normalized against U6 expression. Four independent knockdown experiments were carried out, and triplicate quantitative PCR experiments were done for each condition. Data was Log_2_ transformed and error bars represent the standard deviation.

### Western Blotting

Cells and tissues were lysed in RIPA buffer and stored at −80°C. Lysates were separated on SDS-PAGE gels and electrophoresed onto PVDF membranes. Membranes were probed with antibodies against EZH2 (Epitomics, 1:2000) and tubulin (Calbiochem CP06, 1:2000) in 5% non-fat dry milk in TBST.

### Chromatin Immunoprecipitation

ChIP was carried out using the rabbit polyclonal anti-MYC antibody (Santa Cruz, sc-764), as previously described [26]. Primers are listed in Supplementary Table 1.

### Cell Proliferation Assays

Cells were stained with 0.4% trypan blue, and the number of viable cells was counted using a hemocytometer.

### Luciferase Reporter Assays

The complete and truncated EZH2 3′ UTR were amplified using the primers listed in Table S1. They were cloned into the pMIR-REPORT™ miRNA Expression Reporter Vector System (Ambion) between the HindIII and SpeI sites. The EZH2 promoter reporter vector (S111151) was purchased from SwitchDB. The reporter promoters were transfected along with a Renilla control plasmid, and either non-targeting siRNA, MYC siRNA, miR-26a mimics, or miR-26b mimics. Luciferase activity was measured 24 hours after transfection using the Dual-luciferase reporter assay system (Promega). Three independent experiments were carried out, with 4 replicates for each condition.

### Immunohistochemistry (IHC)

Immunohistochemistry was done with the Power Vision+ poly-HRP IHC Kit (ImmunoVision Inc). Slides were steamed for 40 minutes in EDTA solution (Zymed) and incubated with mouse anti-EZH2 antibody (BD Transduction) overnight. Poly-HRP-conjugated anti-mouse/rabbit IgG antibody was used as secondary antibody. Staining was visualized using 3,3′-Diaminobenzidine (Sigma) and slides were counterstained with hematoxylin.

### Quantitative Image Analysis

Scanned images were analyzed using FriDA as previously described [15].

### Tissues

This study was approved by The Johns Hopkins University School of Medicine institutional review board. Matched normal and tumor pairs were obtained from frozen sections from radical prostatectomy specimens. Specimens comprised Gleason scores 6 and 7, and stages from T2N0Mx to T3BN0Mx (Table S*2*).

### Statistics

Students t-test was performed to determine statistical significance and p-values are indicated in the text. Changes are also indicated in the figures where (*) represents p <0.05.

## RESULTS

### MYC regulates EZH2 in the Lo-MYC murine model of prostate cancer

**Figure 1:**
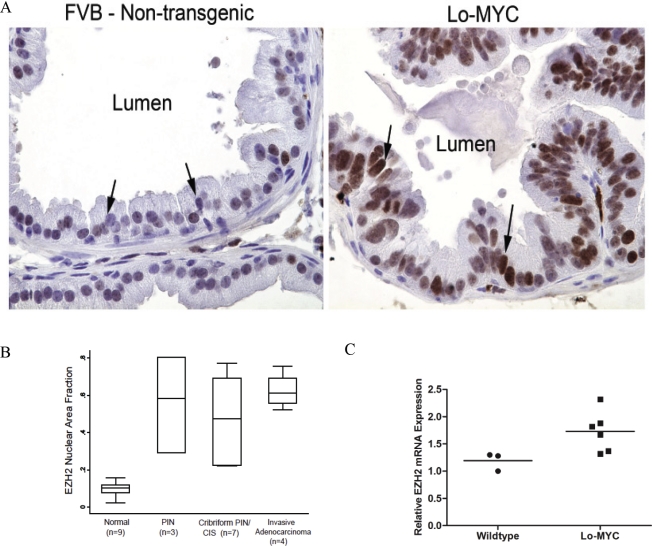
Elevated EZH2 Expression in the Lo-MYC Murine Model of Prostate Cancer (*A*) Increased EZH2 protein expression in the ventral prostates of Lo-MYC mice (right panel), as compared to wild type controls (left panel). (*B*) Semi-quantitative image analysis of EZH2 protein expression in 9 wild type and 14 Lo-MYC mice. EZH2 expression was elevated in PIN, cribriform PIN/CIS, and invasive adenocarcinoma lesions, as compared to normal prostate. (*C*) *EZH2* mRNA is elevated in PIN lesions from the ventral prostates of Lo-MYC (6 mice), as compared to ventral prostates from age-matched wildtype FVB mice (3 mice).

The Lo-MYC transgenic mouse model recapitulates the molecular and histological phenotype of early human prostate cancer formation [24, 27]. In this model, Myc overexpression in the prostate induces the morphologically distinguishing features of PIN, including nuclear and nucleolar enlargement, an increase in mitotic figures, as well as widespread chromatin remodeling. Using these mice for immunohistochemical staining, we observed that the expression of EZH2 protein was barely detectable in control non-transgenic littermate mice, but that levels were markedly upregulated in PIN lesions in the Lo-MYC mice (Fig. 1*A*). Similar to overexpression of Myc, the increased expression of EZH2 protein in PIN lesions was confined to luminal epithelial cells. Semi-quantitative image analysis showed that as compared to wild-type mice, there was a marked increase in expression of EZH2 in PIN lesions, cribriform PIN lesions and early invasive adenocarcinoma lesions in the transgenic mice (Fig. 1*B*). Similar results were obtained in a number of Hi-MYC mice (not shown). Next, used quantitative real time RT-PCR and found that *EZH2* mRNA was also increased in the Myc-overexpressing transgenic mice, compared to the wild type controls (Fig. 1*C*, p<0.03). Thus, in these Myc-driven murine models of prostate cancer, EZH2 elevation occurs downstream of Myc induction.

### MYC and EZH2 are overexpressed in primary prostate cancer specimens

**Figure 2:**
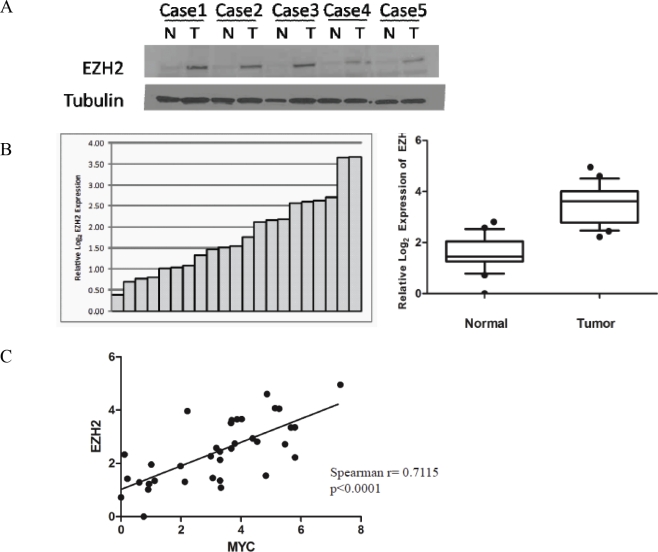
Correlation between MYC and EZH2 expression in primary prostate cancer specimens (*A*) Western blot showing increased EZH2 protein expression in localized prostate cancer specimens, as compared to matched normal tissue. (*B*) Elevated EZH2 mRNA levels in localized prostate cancer specimens, as compared to matched normal tissue, determined by quantitative real-time PCR. (*C*) Positive correlation between the expression of *MYC* and *EZH2* mRNA levels.

It has been shown that EZH2 is overexpressed in prostate cancer, particularly in aggressive metastatic cases [6]. We prepared crude whole cell lysates from 5 matched benign and primary prostate cancer specimens and verified that EZH2 protein expression was elevated in the tumors (Fig. 2*A*). Next, we obtained mRNA from 18 matched normal and primary prostate cancer specimens (Table S*2*) and confirmed that *MYC* and *EZH2* mRNA were indeed overexpressed in the prostate cancers (Wilcoxon signed ranked test, p<0.0002 for both) (Fig. 2*B*, S1*A*). Additionally, in these same specimens there was a significant positive correlation between *MYC* and *EZH2* mRNA expression (Spearman rank correlation coefficient= 0.7115, p<0.0001) (Fig. 2*C*).

### MYC regulates EZH2 mRNA and protein expression in prostate cancer cell lines

**Figure 3:**
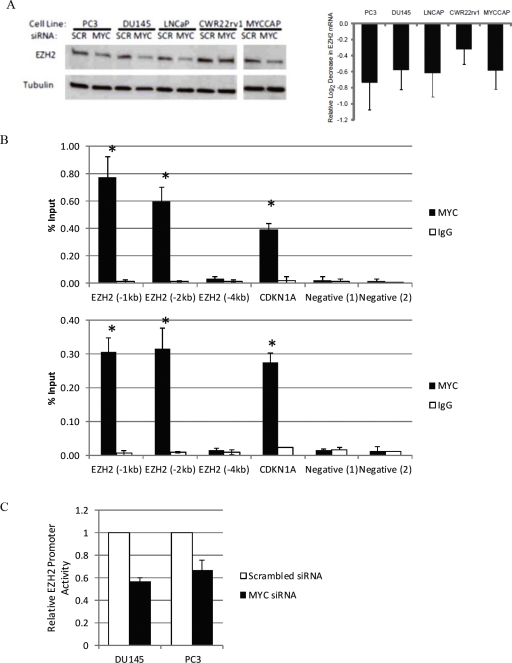
Myc binds to the EZH2 promoter region and activates EZH2 transcription in prostate cancer cell lines (A) Reduced EZH2 protein (Left Panel) and mRNA (Right Panel) expression following siRNA-mediated MYC depletion. (B) Enrichment of Myc binding at the E-box-containing promoter region of EZH2 in 2 prostate cancer cell lines, as determined by ChIP. Immunoprecipitation was carried out with an anti-Myc antibody (black bars) and a control IgG antibody (white bars). (C) Decreased EZH2 promoter activity after MYC depletion (black bars), as compared to control cells (white bars) in human prostate cancer cell lines.

We used siRNA-mediated knockdown to deplete Myc in 4 human prostate cancer cell lines- DU145, PC3, LNCaP and CWR22rv1, as well as in the MYC-CaP cell line, which was derived from the Hi-MYC transgenic mouse model [25]. We have recently shown approximately 50-70% of Myc protein could be reduced using these siRNA transfection conditions in each of these cell lines [28]. 72h after Myc knockdown, we measured the expression of *EZH2* mRNA by quantitative real time PCR, relative to the expression of *TBP*, and found it to be reduced by 30%-60% (p<0.05) (Fig. 3*A*, right panel). Accordingly, as compared to control cells that were transfected with non-targeting siRNA, EZH2 protein was also reduced in cells that had been transfected with siRNA targeting Myc (Fig. 3*A*, left panel). By microarray analyses, we observed an induction of *CDH1* and *ADRB2*, known EZH2 target genes, following MYC depletion in LNCaP and DU145 cells (Fig. S1*B*).

By examining publically available data in the UCSC genome browser database, Myc was observed to associate upstream of the EZH2 transcription start site in K562 human myelogenous leukaemia cells and GM12878 lymphoblastoid cells (Figure S1*C*). To address whether *EZH2* is a direct target of transcriptional activation by Myc in prostate cancer, we carried out chromatin immunoprecipitation (ChIP), and enriched Myc binding approximately 1kb and 2kb upstream of the transcription start site of *EZH2*, which contains a canonical E-box (Figure 3*B*). With increasing distance upstream of the *EZH2* transcription start site (approximately −4kb), there was no significant enrichment of Myc binding. To determine if *EZH2* is a direct MYC target gene, we used firefly luciferase as a reporter of *EZH2* promoter activity. In DU145 and PC3 cells that had been depleted of MYC by siRNA-mediated knockdown, we observed reduced *EZH2* promoter activity (Fig. 5*B*; p<0.02 for both cell lines),indicating that MYC can indeed activate *EZH2* transcription in prostate cancer cell lines.

**Figure 4:**
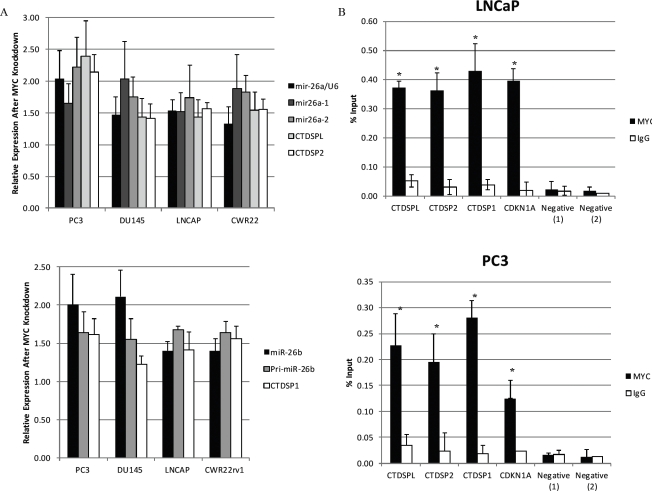

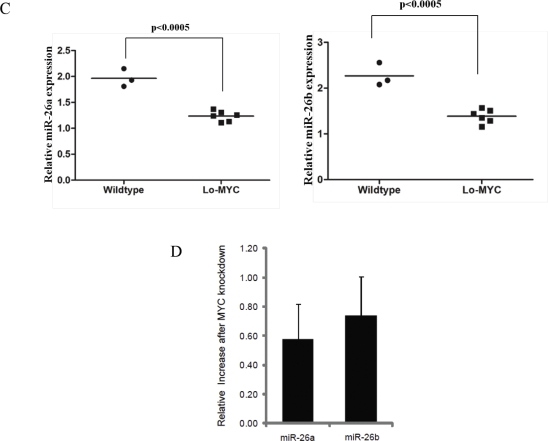
Myc Regulates miR-26a and miR-26b in Human and Murine Prostate Cancer Cells (*A*) (Upper Panel) Induction of miR-26a, its primary forms miR-26a1 and miR-26a2, and the genes in which they are embedded, *CTDSPL* and *CTDSP2*, following siRNA-mediated Myc depletion. (Lower Panel) Induction of miR-26b, its primary form, and the gene in which it is embedded, *CTDSP1*. (*B*) Enriched Myc binding was observed at the promoter regions of *CTDSPL*, *CTDSP2* and *CTDSP1*. Immunoprecipitation was carried out with an anti-Myc antibody (black bars) and a control IgG antibody (white bars). (*C*) Reduced miR-26a (Left Panel) and miR-26b (Right Panel) expression in the Lo-MYC mice, as compared to wildtype controls. (*D*) Induction of miR-26a and miR-26b after Myc depletion in MYC-CaP cells.

**Figure 5:**
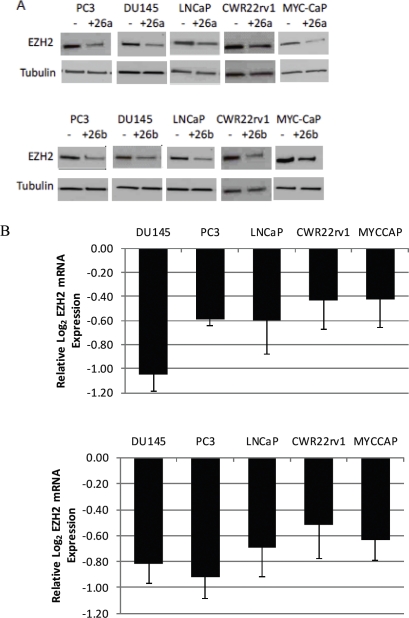

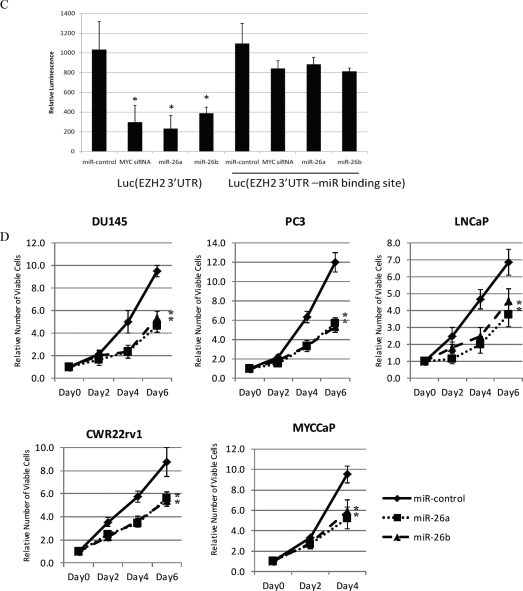
MiR-26a and miR-26b Regulate EZH2 Expression in Human and Murine Prostate Cancer Cells (*A,B*) MiR-26a and miR-26b repress EZH2 protein (*A*) and mRNA (*B*) expression. (*C*) miR-26a and miR-26b bind to the 3′UTR of *EZH2* in prostate cancer cells. (*D*) Reduced proliferation of prostate cancer cell lines after transfection with miR-26a (dotted lines) and miR-26b (dashed lines), as compared to control miR (solid lines).

### Myc represses miR-26a and miR-26b expression

Myc was previously reported to regulate the expression of miRNAs [17], including miR-26a and miR-26b in P493 lymphoma cells, by direct binding to their host Pol II gene promoters (*CTDSPL*, *CTDSP2*, *CTDSP1*). However, such regulation has not been evaluated in prostate cancer. Since regulation of target genes by Myc can be cell type and context specific, we determined whether similar mechanisms were operative in prostate cancer cells. siRNA-mediated MYC depletion in 4 human prostate cancer cell lines was followed by quantitative real-time PCR to measure the expression of the mature miR-26a, its 2 primary forms, mir-26a1, and mir-26a2, as well as the host genes in which they are embedded, *CTDSPL* and *CTDSP2*. Additionally, we measured the expression of the mature and primary forms of miR-26b, and *CTDSP1*, the gene which harbors miR-26b. Following MYC-depletion, we found a coordinate increase in the mRNA levels of *CTDSPL*, *CTDSP2* and *CTDSP1*, and both the mature and primary forms of miR-26a and miR-26b in all 4 cell lines (p<0.02 for all) (Fig. 4*A*). To determine if MYC may be directly regulating miR-26a via repression of *CTDSPL* and *CTDSP2*, and miR-26b via repression of *CTDSP1*, we carried out chromatin immunoprecipitation on LNCaP and PC3 cells. We found a significant enrichment of MYC at the promoters of *CTDSPL*, *CTDSP2* and *CTDSP1* in both prostate cancer cell lines (Fig. 4*B*). MYC was previously reported to associate with these promoters in P493 lymphoma cells [17]. In terms of Lo-MYC mice, we found that levels of both miR-26a and miR-26b were reduced in PIN lesions as compared to normal epithelium in wild type controls (p<0.0005 for both) (Fig. 4*C*). Further, miR-26a and miR-26b expression was increased in MYC-CaP cells after MYC depletion (p<0.02 for both) (Fig. 4*D*). Therefore, the regulation of these miRNAs by MYC occurs in both mouse and human prostatic cancer cells.

### EZH2 is a target of miR-26a and miR-26b in prostate cancer

Previous work in muscle, lymphoma and HeLa cells have shown that miR-26a and miR-26b can negatively regulate EZH2 expression, and that this occurs via binding to the highly conserved predicted miR-26a/b binding site within the 3′ UTR of *EZH2* [21-23]. However, miR-26a did not regulate EZH2 in breast cancer or acute myeloid leukemia cells [5, 29], indicating that miRNA-regulation of target gene expression may be tissue and/or context specific. To determine if mir-26a and miR-26b can target EZH2 in prostate cancer cells, we transfected miR-26a and miR-26b mimics into one mouse and four human prostate cancer cell lines. We observed a reduction in *EZH2* mRNA levels in all 5 cell lines (p<0.05), and a similar decrease in EZH2 protein following transient overexpression of miR-26a and miR-26b (Fig 5*A, B*). In the Lo-MYC mouse model, there was a significant negative correlation between the expression of *EZH2* mRNA and miR-26a (Spearman rank correlation coefficient= −0.8667, p=0.0045), as well as *EZH2* mRNA and miR-26b (Spearman rank correlation coefficient= −0.8167, p=0.0108) (Fig. S2*A*). To verify specific associations between miR-26a and miR-26b and their predicted binding site on the *EZH2* 3′UTR in prostate cancer cells, we cloned the 3′UTR of *EZH2* into a luciferase reporter vector [21]. Co-transfection with either miR-26a mimics, miR-26b mimics or Myc siRNA resulted in reduced reporter activity, as compared to transfection with a non-targeting miR (p<0.03) (Fig. 5*C*). As an additional control, we generated a luciferase reporter vector with a truncated *EZH2* 3′UTR, which lacks the miR-26a/b binding site. Using this construct, there was no significant reduction in reporter activity following co-transfection with miR-26a mimics, miR-26b mimics or Myc siRNA (Fig. 5*C*). There was also was an induction of *CDH1* and *ADRB2*, known EZH2 target genes, following miR-26a and miR-26b overexpression in LNCaP cells (Fig. S2*B*).

To determine the biological relevance of miR-26a and miR-26b in prostate cancer cells, we assessed the effect of miR-26a and miR-26b transfection on the proliferation of 4 human prostate cancer cell lines and the Myc-driven mouse prostate cancer cell line. In all cell lines, proliferation was suppressed (p<0.01) (Fig. 5*D*). This suggests that Myc-mediated repression of miR-26a and miR-26b may be an important factor in maintaining the proliferative capacity of prostate cancer cells.

### MYC, EZH2 and miR-26a expression in localized prostate cancer samples

**Figure 6:**
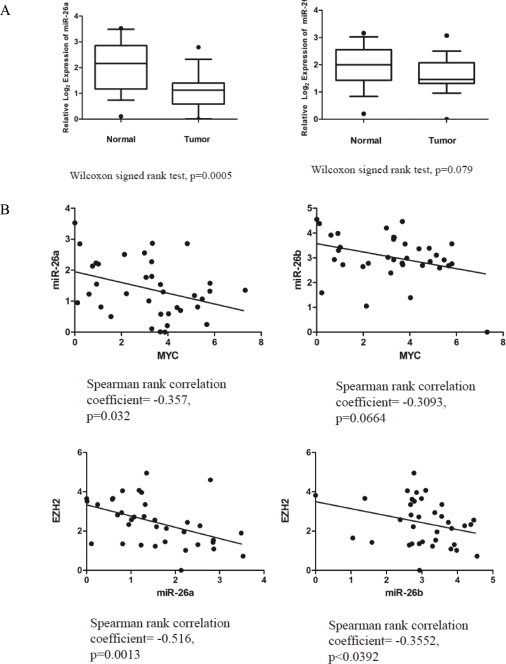
MiR-26a Expression in Primary Prostate Cancer Specimens (*A*) Reduced miR-26a expression in primary prostate cancer cases, normalized to matched benign prostate tissue. Inverse correlations were observed between *MYC* and miR-26a/b (*B*), as well as miR-26a/b and *EZH2* (*C*).

Next, to determine the relevance of miR-26a and miR-26b in clinical prostate cancer, we measured their expression in 18 matched normal and primary prostate cancer specimens (Table S*2*) and found both to be downregulated in cancer in most cases (Wilcoxon signed rank test, p=0.0005 for miR-26a, p=0.079 for miR-26b) (Fig. 6*A*, Fig. S*3A*). There was a significant inverse correlation between the expression of *MYC* and miR-26a (Spearman rank correlation coefficient= −0.357, p=0.032), while that for *MYC* and miR-26b did not achieve statistical significance (Fig. 6*B*, Fig. S*3B*). We also observed significant inverse correlations between miR-26a and *EZH2* mRNA (Spearman rank correlation coefficient= −0.516, p=0.0013), and miR-26b and *EZH2* mRNA (Spearman rank correlation coefficient= −0.3552, p<0.0392) (Fig. 6*C*, Fig. S*3C*).

## DISCUSSION

EZH2 mRNA and protein levels are elevated during progression of a number of types of cancer, including breast and prostate cancer [6, 30-32]. Further, several studies have shown oncogenic activity for EZH2 in a range of tumor cell types [2, 9, 33]. Prior to the present study, three molecular mechanisms were identified that could be responsible for overexpression of EZH2 in prostate cancer--amplification of the *EZH2* gene [8], the deletion of its negative regulator miR-101 [5], and transcriptional regulation of *EZH2* by ETS gene family members [12, 13]. Interestingly, two of these mechanisms (EZH2 amplification and miR-101 deletion) are encountered more frequently in advanced and castrate resistant prostate cancers than in primary untreated prostate cancers, and ETS family gene fusions and ERG protein overexpression are rarely seen in isolated PIN lesions or PIN lesions associated with ERG-negative carcinomas [34]. While EZH2 elevation is most marked in advanced/castrate resistant metastatic lesions, EZH2 is also very commonly overexpressed in almost all human PIN and early prostate cancer lesions [6]. Whether EZH2 overexpression is also regulated by other mechanisms in PIN and primary prostate cancer lesions has not previously been examined. Here, we show that Myc induces overexpression of EZH2 by two independent yet complementary mechanisms, and that this overexpression is seen in PIN lesions in Lo-MYC and Hi-MYC mice, as well as in human tissue samples. Since Myc is commonly overexpressed in human PIN and early carcinoma lesions, it is likely that overexpression of Myc is a key driver of EZH2 overexpression during the early phases of prostate cancer formation. Additionally, since Myc is also overexpressed, and commonly shows elevated gene copy number in advanced/hormone refractory prostate cancers, these results also provide additional mechanisms by which EZH2 overexpression may occur and/or be maintained at this stage of the disease.

Using 5 prostate cancer cell lines (4 human, 1 mouse), we showed that Myc depletion by siRNA results in decreased EZH2 mRNA and protein. It was previously reported that p53 can suppress *EZH2* [35], and that E2F can induce *EZH2* [36], but little is otherwise known about the direct transcriptional regulation of *EZH2*. Using ChIP, we determined in 2 prostate cancer cell lines that MYC directly associates with the E-box-containing promoter region of *EZH2*. While this manuscript was in process, Salvatori et al. reported similar findings in acute myeloid leukemia, showing that Myc directly binds upstream of EZH2 and activates its transcription [29]. While EZH2 expression was shown to regulate MYC expression in a glioma tumor model [37], it is clear in the Lo-MYC mouse model that MYC is elevated prior to EZH2 overexpression.

Since Myc is known to regulate miRNAs, in addition to mRNAs, we sought to determine Myc -responsive miRNAs in prostate cancer. MiR-26a and miR-26b have been reported to be Myc -repressed in lymphoma cells [17, 21]. By quantitative real-time PCR, we found that Myc represses miR-26a and miR-26b in all prostate cancer cell lines studied. Additionally, we observed decreased miR-26a and miR-26b expression in the Lo-MYC mice, as compared to the wildtype controls. In LNCaP and PC3 cells, we observed an enrichment of Myc binding at the promoter regions of *CTDSPL*, *CTDSP2* and *CTDSP1*, genes whose introns harbor the miR-26a and miR-26b primary transcripts. EZH2 is a reported target of miR-26a in muscle and lymphoma cells [21, 22], and of miR-26b in HeLa cells [23]. In prostate cancer cells, we found that both miR-26a and miR-26b repressed both EZH2 mRNA and protein. Further, using heterologous reporter constructs we found that the conserved miR-26a/b binding site within the 3′ UTR of EZH2 was required for this repression. As such, Myc may contribute to EZH2 elevation in prostate cancer, by directly activating *EZH2* transcription, and by repressing its negative regulators, miR-26a and miR-26b. Previously, Cao et al. reported that overexpression of miR-26a reduced EZH2 expression in DU145, but not PC3 or LNCaP cells, even though miR-26a inhibited the expression of a reporter construct containing the 3′UTR of EZH2 in all three cell lines [38]. This somewhat discrepant finding from ours may be a result of different transfection conditions or time points at which samples were harvested.

In our set of matched primary prostate cancer specimens and normal prostate tissue, we found *MYC* and *EZH2* elevation, and a positive correlation between the expression of the two genes. EZH2 was reported to positively regulate MYC in glioblastoma cells [37]. Hence, this may set up a feed-forward loop that sustains high levels of Myc and EZH2, thus deregulating these central regulatory nodes and promoting transformation, and contributing to the oncogenic phenotype.

Reduced miR-26a expression has been noted in various cancers, including hepatocellular carcinoma, thyroid anaplastic carcinoma, rhabdomyosarcoma and clear cell renal cell carcinoma [39-44]. However, miR-26a has been reported to be amplified in gliomas, and to promote gliomagenesis [43]. Previous miRNA profiling studies in prostate cancer have reported both the down-regulation [45, 46], and the up-regulation of miR-26a [47-49]. Decreased miR-26b expression has been reported in liver, head and neck cancers, and in hormone refractory prostate cancer [46, 50, 51], while its upregulation was reported in bladder cancer [52]. Thus, whether miR-26a and miR-26b facilitate oncogenesis, or act as a tumor suppressor, may depend on the cellular context.

By quantitative real-time PCR, we found that the expression of miR-26a was reduced in a high fraction of localized prostate cancer, and correlated negatively with the expression of both *MYC* and *EZH2*. In general, miR-26b expression was also reduced in the tumors, though this reduction was not as consistent. Transient transfection of miR-26a and miR-26b mimics into 5 prostate cancer cell lines resulted in suppressed proliferation, indicating that these MYC targets of repression may have tumor-suppressive functions in the context of prostate cancer. In a murine liver cancer model, in which a similar under-expression of miR-26a was observed, targeted delivery of miR-26a showed a therapeutic effect [40]. Thus, similar strategies may be applicable to the treatment of prostate cancer.

**Figure 7:**
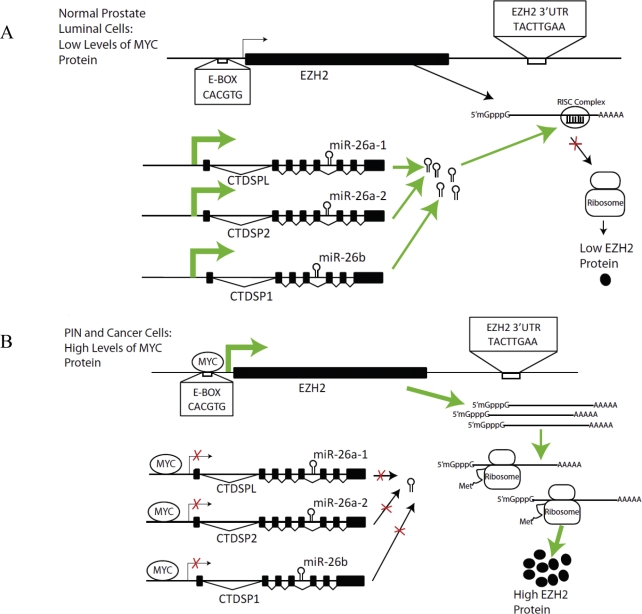
Model of Regulation of EZH2 by Myc by Two Distinct Mechanisms in Prostate Cancer (A) In normal prostate luminal cells, Myc protein expression is low. Myc-induced EZH2 transcription occurs at a low level, resulting in basal expression levels of EZH2. Since MYC represses the transcription of CTDSPL, CTDSP2 and CTDSP1, when MYC levels are low, these genes, which harbor miR-26a and miR-26b, are actively transcribed. MiR-26a and miR-26b are incorporated into the RISC complex and bind specifically to their complementary site on the 3′ UTR of EZH2, destabilizing EZH2 mRNA and repressing its translation. As such, EZH2 is maintained at a low level by transcriptional and post-transcriptional regulatory mechanisms. (B) In prostate cancer, elevated Myc levels drive EZH2 overexpression. MYC binds to the E-box-containing promoter region of EZH2 and activates its transcription. Concurrently, MYC represses CTDSPL, CTDSP2 and CTDSP1, in which miR-26a and miR-26b are embedded. As such, miRNA-mediated post-transcriptional silencing of EZH2 mRNA is reduced. This results in elevated EZH2 protein expression.

In summary, we report that Myc can activate EZH2 expression in prostate cancer by 2 distinct mechanisms-via direct transcriptional activation of the EZH2 promoter, as well as via repression of miR-26a and miR-26b, which themselves can repress EZH2. The frequent and early overexpression of Myc in PIN and primary prostate cancer cases may account in part for the common upregulation of EZH2 in these lesions. A model for how Myc regulates EZH2 is shown in Fig. 7. First, Myc upregulates EZH2 directly by binding to the *EZH2* promoter and stimulating its transcription. Second, by simultaneously binding to and inhibiting transcription of the host genes encoding miR-26a and miR-26b, Myc enforces this upregulation by inhibiting post-transcriptional silencing of *EZH2* mRNA. Thus, by binding to 4 separate loci and affecting both activation and repression, Myc coordinates the upregulation of EZH2 protein.

These findings have additional implications for the transformation of prostate epithelial cells. For example, it is quite plausible that Myc may stimulate prostate cancer initiation, in part via EZH2-mediated chromatin remodeling and gene silencing. Hence, these results support the concept that EZH2 may be an early contributor to prostate cancer development [53], in addition to being a potent driver of the progression to aggressive, metastatic disease. EZH2 can modulate the expression of numerous regulatory networks, and there is significant enrichment for EZH2-mediated H3K27me3 polycomb repression signatures in embryonic stem cells, embryonic fibroblasts and advanced prostate cancer [4, 13, 54]. Myc has also been shown to activate an embryonic stem cell-like module of gene expression and can prevent the terminal differentiation of prostate cancer cells (Koh et al., in preparation). Hence, Myc overexpression may drive prostate cancer initiation by perturbing normal prostate differentiation programs and aberrantly activate embryonic stem-cell like programs, in part by the induction of EZH2.

## Supplementary Table and Figures


