# Supplementary material for: Myc Enforces Overexpression of EZH2 in Early Prostatic Neoplasia via Transcriptional and Post-transcriptional Mechanisms
Source: Oncotarget. 2011 Sep 19;2(9):669–83. doi: 10.18632/oncotarget.327 (PMC3248223; doi:10.18632/oncotarget.327)

**Supplementary Table 1.** Primers used for real-time quantitative PCR and cloning. Primers were previously described by Cao et al. (2008), Chang et al. (2008), Chen et al. (2005), Richter et al. (2009), Suzuki et al. (2009), Ushizawa et al. (2007) and Yu et al. (2010), or designed using Primer3 software.

| | |
|---|---|
| EZH2chip1-F<br>EZH2chip1-R | 5'-GACACGTGCTTAGAACTACGAACAG-3'<br>5'-TTTGGCTGGCCGAGCTT-3' |
| EZH2chip2-F<br>EZH2chip2-R | 5'-CCAACATTGGAGTGATTCAG-3'<br>5'-TCATCAGATGATTTAGCCCA-3' |
| EZH2chip3-F<br>EZH2chip3-R | 5'-ATCCAGCCCCAAGCTGTTT-3'<br>5'-GAACATGAGGTGGTGATAAAAATAAGG-3' |
| CTDSP1chipF<br>CTDSP1chipR | 5'-CAAAATAGTAACGACGAGTGAAAAGAA-3'<br>5'-TGGTCTTTTTCCTCGTTTATGAAGTT-3' |
| CTDSP2chip-F<br>CTDSP2chip-R | 5'-CTCCATCTGTGAGCGGCC-3'<br>5'-AAAATAGCAAAGCTCCCGACTG-3' |
| CTDSPLchip-F<br>CTDSPLchip-R | 5'-GGAGAGACTGGGAGCGAGTGT-3'<br>5'-CAAACTCACAACCTCCCGGT-3' |
| EZH2-F<br>EZH2-R | 5'-GCCAGACTGGGAAGAAATCTG-3'<br>5'-TGTGCTGGAAAATCCAAGTCA-3' |
| CTDSP1-F<br>CTDSP1-R | 5'-GGCAAAGGTGACCAGAAGTC-3'<br>5'-TCAGGGAGCAGGTATTGGAC-3' |
| CTDSP2-F<br>CTDSP2-R | 5'-GGCCTGGTGTCCAAGTCCT-3'<br>5' CAGAAAAGGGCCTTGAAGATGT 3' |
| CTDSPL-F<br>CTDSPL-R | 5'-CTGGCTGCCTCTGTTCCTACT-3'<br>5'-TCAAATGTCCAGCAGAAATCCA-3' |
| primir26a1-F<br>primir26a1-R | 5'-GCCCAATGGCATAGCAAGA-3'<br>5'-GGCCAGTCATGCTTACAGTCAC-3' |
| primir26a2-F<br>primir26a2-R | 5'-AGGCATCCCTGTGATGAAGGT-3'<br>5'-TTGAGAGCTCCTTGCTGCTGT-3' |
| primir26b-F<br>primir26b-R | 5'-CTCAAGGGCTTGTGCTGACT-3'<br>5'-ACCTCAGCCTAGTGCAGGAA-3' |
| CDH1-F<br>CDH1-R | 5'-TGGAGAGACACTGCCAACTG-3'<br>5'-AGGCTGTGCCTTCCTACAGA-3' |
| Cloning-<br>EZH2.UTR-F<br>EZH2.UTR-Rfull<br>EZH2.UTR-Rtruncated | <br>5'-CGCTACTAGTCTTGACATCTGCTACCTCCTC-3'<br>5'-CAGAAGCTTACAAGTTCAAGTATTCTTTA-3'<br>5'-CAGAAGCTGTACCATACTGCATTATTGCAAAAATTC-3' |

**Supplementary Table 2.** Pathologic features of radical prostatectomy cases used in this study.

| Gleason Sum | Pathological Stage | | | |
|---|---|---|---|---|
| | T2N0MX | T3AN0MX | T3BN0MX | Total |
| 6 | 2 | 1 | 0 | 3 |
| 7 | 2 | 10 | 3 | 15 |
| Total | 4 | 11 | 3 | 18 |

**Supplementary Figure 1.** (*A*) Relative *MYC* and *EZH2* expression levels in primary prostate cancer tissues, normalized to their expression in matched normal prostate tissue from the same patient. (*B*) Increased *CDH1* and *ADRB2* expression after siRNA-mediated MYC depletion in LNCaP and DU145 cells. (*C*) MYC binding to EZH2 in GM12878 and K562 cells using hg18 genome assembly, obtained from publicly available data at http://genome.ucsc.edu.

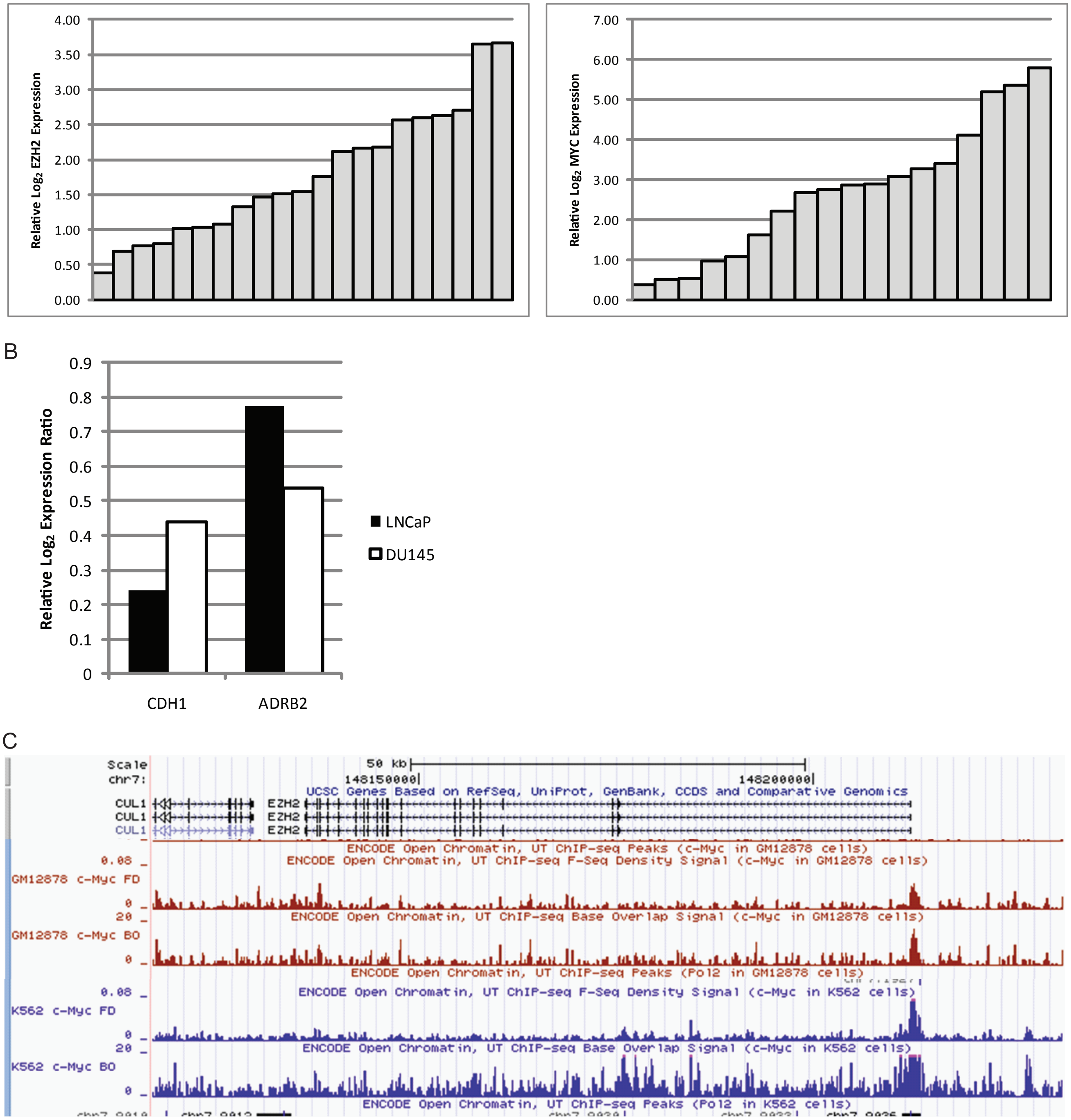

**Supplementary Figure 2.** (*A*) Negative correlation between *EZH2* and miR-26a, and *EZH2* and miR-26b expression in Lo-MYC and wildtype mice. (*B*) Increased *CDH1* and *ADRB2* expression in LNCaP cells after miR-26a and miR-26b transfection.

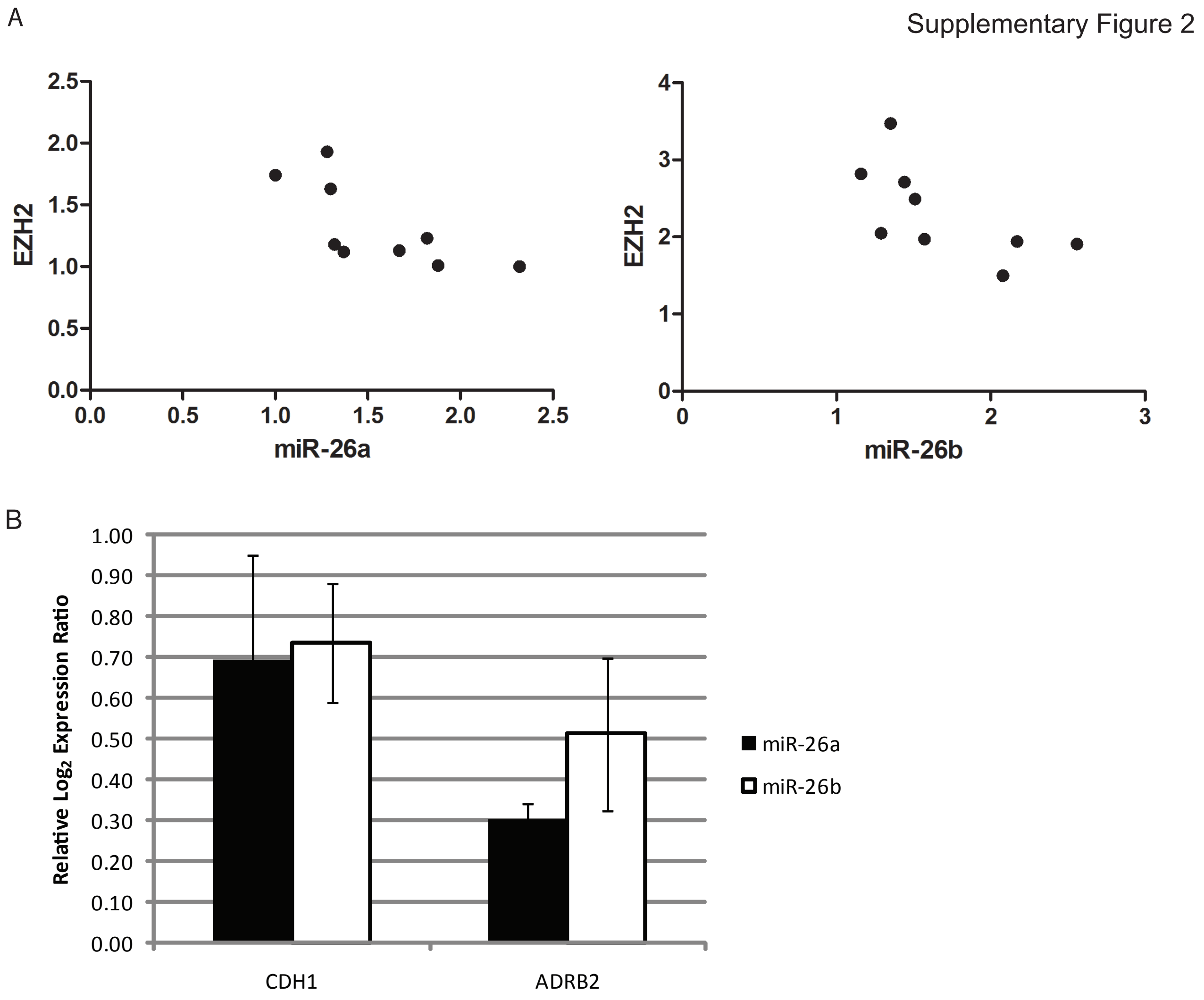

**Supplementary Figure 3.** (*A*) Relative miR-26b expression levels in matched normal and primary prostate cancer tissues. (*B*) Negative correlation between miR-26b and *MYC*. (*C*) Negative correlation between miR-26b and *EZH2*.

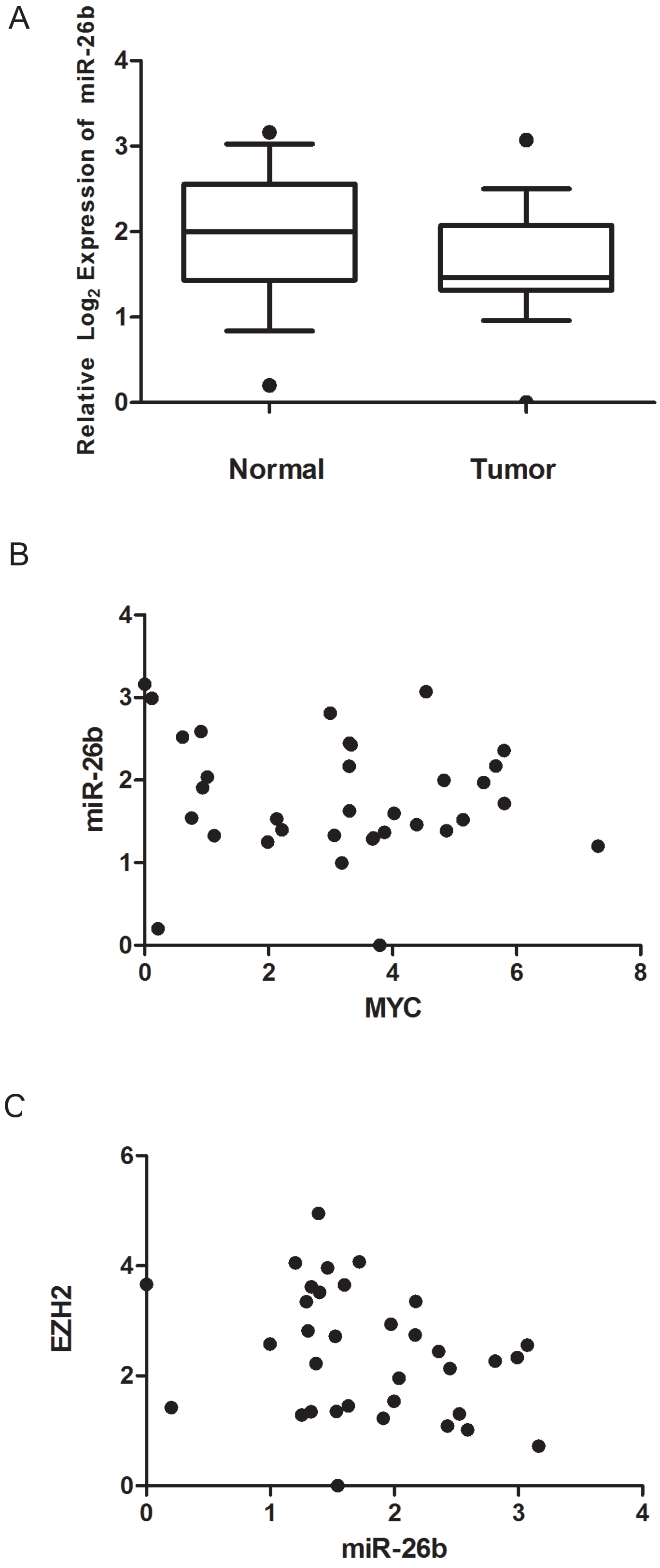

Supplement: Supplementary file 1 [file oncotarget-02-669-s001.pdf]
